# Supplementary material for: Helix breaking transition in the S4 of HCN channel is critical for hyperpolarization-dependent gating
Source: eLife. 2019 Nov 27;8:e53400. doi: 10.7554/eLife.53400 (PMC6904216; doi:10.7554/eLife.53400)
Supplement: Supplementary file 1. [file elife-53400-supp1.pdf]

Supplementary Table 1

ΔG_r(HCN1→mut) [kcal/mol]	ΔG_a(HCN1→mut) [kcal/mol]
-15.4 ± 0.3	-15.0 ± 0.2
-15.3 ± 0.2	-14.5 ± 0.2
-15.4 ± 0.4	-14.9 ± 0.2
-	-14.4 ± 0.4
-	-14.5 ± 0.2
