# Supplementary material for: Helix breaking transition in the S4 of HCN channel is critical for hyperpolarization-dependent gating
Source: eLife. 2019 Nov 27;8:e53400. doi: 10.7554/eLife.53400 (PMC6904216; doi:10.7554/eLife.53400)
Supplement: Transparent reporting form [file elife-53400-transrepform.docx]

***eLife’s* transparent reporting form**

We encourage authors to provide detailed information *within their submission* to facilitate the interpretation and replication of experiments. Authors can upload supporting documentation to indicate the use of appropriate reporting guidelines for health-related research (see [EQUATOR Network](http://www.equator-network.org/%20)), life science research (see the [BioSharing Information Resource](https://biosharing.org/)), or the [ARRIVE guidelines](http://www.plosbiology.org/article/info:doi/10.1371/journal.pbio.1000412) for reporting work involving animal research. Where applicable, authors should refer to any relevant reporting standards documents in this form.

If you have any questions, please consult our Journal Policies and/or contact us: [editorial@elifesciences.org](mailto:editorial@elifesciences.org).

**Sample-size estimation**

- You should state whether an appropriate sample size was computed when the study was being designed
- You should state the statistical method of sample size computation and any required assumptions
- If no explicit power analysis was used, you should describe how you decided what sample (replicate) size (number) to use

Please outline where this information can be found within the submission (e.g., sections or figure legends), or explain why this information doesn’t apply to your submission:

Molecular dynamics simulations were used as a sampling method; 2 runs 18.8 and 22.8 us long have been performed for the M153T/I160V HCN1 mutant. Each voltage sensor domain of the channel was considered as an independent data point; therefore, we have obtained 8 datapoints from the simulations in total. The number of runs and their duration were limited by the granted amount of recourses on Anton supercomputer.
For the calculations of the gating charge and the coupling function: 10 conformations of the resting and activated states (5 for each) were used; for every conformation 9 short MD simulations were performed.

For the free energy estimation: 3 and 5 conformations of the resting and activated states, respectively, were considered.

This information is reported in the Methods section.

As is standard in the field, sample size of five and above were used for electrophysiological characterization each of the mutants.

**Replicates**

- You should report how often each experiment was performed
- You should include a definition of biological versus technical replication
- The data obtained should be provided and sufficient information should be provided to indicate the number of independent biological and/or technical replicates
- If you encountered any outliers, you should describe how these were handled
- Criteria for exclusion/inclusion of data should be clearly stated
- High-throughput sequence data should be uploaded before submission, with a private link for reviewers provided (these are available from both GEO and ArrayExpress)

Please outline where this information can be found within the submission (e.g., sections or figure legends), or explain why this information doesn’t apply to your submission:

2 independent MD runs were performed.

For the calculations of the gating charge and the coupling function: 90 short MD simulations were performed for the resting and activated states in total.

For the free energy estimation: 8 FEP transitions were computed.

This information is reported in the Methods section; the results of the runs are reported in the Results section and the Supplementary Information. All the data is included in the manuscript.

Experimental details about the size of the sample is included in figure legends.

**Statistical reporting**

- Statistical analysis methods should be described and justified
- Raw data should be presented in figures whenever informative to do so (typically when N per group is less than 10)
- For each experiment, you should identify the statistical tests used, exact values of N, definitions of center, methods of multiple test correction, and dispersion and precision measures (e.g., mean, median, SD, SEM, confidence intervals; and, for the major substantive results, a measure of effect size (e.g., Pearson's r, Cohen's d)
- Report exact p-values wherever possible alongside the summary statistics and 95% confidence intervals. These should be reported for all key questions and not only when the p-value is less than 0.05.

Please outline where this information can be found within the submission (e.g., sections or figure legends), or explain why this information doesn’t apply to your submission:

For Figure 1C, D and H, Figure 3B, and Figure 6B the data is shown as boxplots with median, 25-75% (box), and 1-99% (bars). For Figure 1F and G, the supplementary table 1, the data is shown as mean and standard error. For Figure 2C and Figure S3A, the data is shown as mean. Figure 1A and S2A shows the raw data.

The information is provided in the figure legends and Methods section.

(For large datasets, or papers with a very large number of statistical tests, you may upload a single table file with tests, Ns, etc., with reference to sections in the manuscript.)

**Group allocation**

- Indicate how samples were allocated into experimental groups (in the case of clinical studies, please specify allocation to treatment method); if randomization was used, please also state if restricted randomization was applied
- Indicate if masking was used during group allocation, data collection and/or data analysis

Please outline where this information can be found within the submission (e.g., sections or figure legends), or explain why this information doesn’t apply to your submission:

N/A

**Additional data files (“source data”)**

- We encourage you to upload relevant additional data files, such as numerical data that are represented as a graph in a figure, or as a summary table
- Where provided, these should be in the most useful format, and they can be uploaded as “Source data” files linked to a main figure or table
- Include model definition files including the full list of parameters used
- Include code used for data analysis (e.g., R, MatLab)
- Avoid stating that data files are “available upon request”

Please indicate the figures or tables for which source data files have been provided:

The raw data (MD simulations) will be available through Anton supercomputer website. The refined structure of HCN1, and the multiple sequence alignments of the EAG and HCN families (the S4 segment) are included as a part of the Supplementary Information.
